# A methylation-based nomogram for predicting survival in patients with lung adenocarcinoma

**DOI:** 10.1186/s12885-021-08539-4

**Published:** 2021-07-12

**Authors:** Xuelong Wang, Bin Zhou, Yuxin Xia, Jianxin Zuo, Yanchao Liu, Xin Bi, Xiong Luo, Chengwei Zhang

**Affiliations:** 1 Department of Thoracic Surgery, Capital Medical University Electric Power Teaching Hospital, Beijing, 100073 China; 2 Department of emergency, Capital Medical University Electric Power Teaching Hospital, Beijing, 100073 China; 3 Department of Internal Medicine, Beijing Nuclear Industry Hospital, Beijing, 100822 China

**Keywords:** Lung adenocarcinoma, DNA methylation, Differentially methylated sites, Prognosis, Signature

## Abstract

**Background:**

DNA methylation alteration is frequently observed in Lung adenocarcinoma (LUAD) and may play important roles in carcinogenesis, diagnosis, and prognosis. Thus, this study aimed to construct a reliable methylation-based nomogram, guiding prognostic classification screening and personalized medicine for LUAD patients.

**Method:**

The DNA methylation data, gene expression data and corresponding clinical information of lung adenocarcinoma samples were extracted from The Cancer Genome Atlas (TCGA) database. Differentially methylated sites (DMSs) and differentially expressed genes (DEGs) were obtained and then calculated correlation by pearson correlation coefficient. Functional enrichment analysis and Protein-protein interaction network were used to explore the biological roles of aberrant methylation genes. A prognostic risk score model was constructed using univariate Cox and LASSO analysis and was assessed in an independent cohort. A methylation-based nomogram that included the risk score and the clinical risk factors was developed, which was evaluated by concordance index and calibration curves.

**Result:**

We identified a total of 1362 DMSs corresponding to 471 DEGs with significant negative correlation, including 752 hypermethylation sites and 610 hypomethylation sites. Univariate cox regression analysis showed that 59 DMSs were significantly associated with overall survival. Using LASSO method, we constructed a three-DMSs signature that was independent predictive of prognosis in the training cohort. Patients in high-risk group had a significant shorter overall survival than patients in low-risk group classified by three-DMSs signature (log-rank *p* = 1.9E-04). Multivariate cox regression analysis proved that the three-DMSs signature was an independent prognostic factor for LUAD in TCGA-LUAD cohort (HR = 2.29, 95%CI: 1.47–3.57, *P* = 2.36E-04) and GSE56044 cohort (HR = 2.16, 95%CI: 1.19–3.91, *P* = 0.011). Furthermore, a nomogram, combining the risk score with clinical risk factors, was developed with C-indexes of 0.71 and 0.70 in TCGA-LUAD and GSE56044 respectively.

**Conclusions:**

The present study established a robust three-DMSs signature for the prediction of overall survival and further developed a nomogram that could be a clinically available guide for personalized treatment of LUAD patients.

**Supplementary Information:**

The online version contains supplementary material available at 10.1186/s12885-021-08539-4.

## Background

Lung cancer is the leading cause of cancer-related deaths worldwide [1], including two main types known as small-cell lung carcinoma (SCLC) and non-small-cell lung carcinoma (NSCLC). Lung adenocarcinoma (LUAD) is the most predominant subtype of NSCLC, with increased incidence over the past decades worldwide [2]. Despite recent advances in surgical techniques, radiotherapeutic interventions and combined chemotherapy strategies, the long-term survival rate of patients diagnosed with LUAD has not significantly improved [3]. Thus, it is indeed urgent to identify specific details regarding characteristic molecules in LUAD tissue to evaluate the prognosis of LUAD and develop strategies for personalized therapy.

DNA methylation, as the key element in epigenetic modifications, plays a significant role in the regulation of cellular functions and carcinogenesis. Increasing studies demonstrated that epigenetic alterations in DNA methylation were relevant to the progression and metastasis of LUAD [4–7]. Shen et al. demonstrates that the methylation status of homeobox A9 (*HOXA9*), keratin-associated protein 8–1 (*KRTAP8–1*), cyclin D1 (*CCND1*), and tubby-like protein 2 (*TULP2*) has great potential for the early recognition of LUAD in the undetermined lung nodules [8]. Seok et al. found that TGFBI promoter methylation is associated with poor prognosis in lung adenocarcinoma patients [9]. Furthermore, a prognostic DNA methylation signature was established by Sandoval et al. to distinguished patients with high- and low-risk early stage NSCLC, guiding the adjuvant chemotherapy [10]. Additionally, researchers suggested an internal CpG-based signature for survival prediction of lung adenocarcinoma patients. These researches demonstrated that the methylation level is deemed a crucial molecular biomarker for the diagnosis and prognosis of LUAD patients [11–13]. However, limited by either the current expertise on the association between the epigenetic modifications and clinical outcomes or lack of independent validation as small sample size, the identification of a robust prognostic DNA methylation signature is of considerable importance for LUAD patients.

In the present study, we extracted the DNA methylation data, gene expression data and corresponding clinical information of lung adenocarcinoma samples from The Cancer Genome Atlas (TCGA) database to select the differentially methylated sites (DMSs) corresponding to dysregulated genes and further explore the biological processes in which the aberrant methylation genes might be involved. Moreover, performing univariate Cox and LASSO analysis, we constructed a robust DMSs-based prognostic signature and validated the prognostic performance in an independent cohort extracted from Gene Expression Omnibus (GEO). Furthermore, combing DMSs-based prognostic signature with clinical risk factors, we constructed a nomogram that could provide insight into regarding survival prediction and serve as a clinically available guide for personalized treatment of LUAD patients.

## Methods

### Data processing

All datasets and clinical information were described in Table 1 and Supplementary Table S1. The DNA methylation data (459 LUAD tissues and 30 normal tissues) and gene expression data (513 LUAD tissues and 59 normal tissues) of lung adenocarcinoma samples were extracted from TCGA (https://cancergenome.nih.gov/). Methylation beta-values derived from Illumina Infinium Human Methylation 450 BeadChip platform were extracted as site methylation measurements. The normalized count values of level 3 gene expression data derived from Illumina HiSeqV2 were extracted as gene expression measurements. Clinical information of 513 LUAD patients was obtained from TCGA. After corresponding patients with both methylation data and expression data, ninety-six LUAD patients were excluded because of unknown survival time, age, and tumor stage. Ultimately, 417 patients were retained in our study. An independent dataset (GSE56044 [14]) collected from GEO (https://www.ncbi.nlm.nih.gov/geo/) was used to test the prognostic ability, containing 82 LUAD patients with both methylation data and clinical information.

**Table 1:** Cohorts analyzed in present study

	Training cohort (TCGA-LUAD)			Validation cohort (GSE56044)	
	Methylation data	Expression data	Clinical data	Methylation data	Clinical data
Normal	30	59	–	–	–
Tumor	417	417	417	82	82
Platform	Illumina HM450	Illumina HiSeqV2	–	Illumina HM450	–

### Identification of differentially methylated sites

The differentially expressed genes (DEGs) were firstly selected between tumor and normal tissues using edgeR package in R. Multiple test corrections was performed using Benjamini & Hochberg’s method and the cutoff values were set at the FDR < 0.05 and |log2FC| > 2. Then, the methylation sites corresponding to these DEGs were selected. For each methylation site, we test the difference in methylation level between tumor and normal tissues to select differentially methylated site (DMS) by T-test with *p* < 0.05. More importantly, pearson correlation analysis was performed to calculate the correlation between the methylation level of DMS and expression level of corresponding DEG. Such DMSs with significant negative correlation, which were thought to deeply influence the expression of corresponding DEGs, were selected for subsequent analysis.

### Functional enrichment analysis

Functional annotations of DEGs containing DMSs were performed using The Database for Annotation, Visualization and Integrated Discovery (DAVID, https://david.ncifcrf.gov/), which enriched gene oncology and pathways. Three categories, including biological processes, molecular function and cellular components, were involved in Gene oncology (GO). Kyoto Encyclopedia of Genes and Genomes (KEGG, https://www.kegg.jp/) was used to carry out the pathway enrichment, which is an essential database resource for a deep understanding of functions and biological process from large-scale molecular cohorts produced by high-throughput experimental technology. The criterion for significant enrichment was *p* < 0.05.

### Protein-protein interaction (PPI) network

To further explore the interaction among the DEGs, the Search Tool for the Retrieval of Interacting Genes (STRING, http://string-db.org/), a database containing all known and predicted protein interactions, was used to identify a PPI network of DEGs. Each interaction was evaluated by combined score ranged from 0 to 1. The higher the combined score, the more reliable the interaction. In present study, we used a strict combined score > 0.7 as the cut-off criterion to identify reliable interactions among the DEGs. The PPI network was visualized by Cytoscape software (version 3.7.0; www.cytoscape.org). Furthermore, the hub genes in PPI network were extracted using the cytoHubba application.

### Construction of DMSs-based prognostic signature

The univariate Cox regression analysis was firstly performed to calculate the association between the methylation level of each DMS and patient’s overall survival (OS) in training cohort. Those sites with *P*-values less than 0.05 were identified as prognosis-related DMSs. Then, using LASSO method to screen the prognosis-related DMSs and obtain an optimal model subsequently, the prognosis-related DMSs with coefficient not equal to 0 were retained as significant variables and a risk scoring model was established using the combination of weighted methylation values. The risk scores were calculated as shown in the following equation: Risk score = methylation of site 1 * β1 + methylation of site 2 * β2 + …methylation of site n * βn. βi is the regression coefficient of site i, which represents the contribution of site i to the prognostic risk score. Based on the equation, risk scores were calculated for LUAD patients in each cohort. Using the median risk score as the cutoff point, patients were divided into low-risk (risk score below the median value) or high-risk (risk score above the median value) group correspondingly.

### Development of DMSs-based nomogram

To translate the prognostic value of DMSs-based signature into clinical application, a nomogram, including the risk score and the clinical risk factors of LUAD patients evaluated by multivariate Cox proportional-hazards regression, was developed for predicting the 3- and 5-years OS in TCGA-LUAD cohort. The discriminatory ability of the nomogram was evaluated by calculating the concordance index (C-index), which is a measure of discrimination. Calibration plots were plotted to compare the observed and predicted probabilities for the nomogram.

### Statistical analysis

The multivariate Cox proportional-hazards regression model was used to evaluate the independent prognostic value of the signature after adjusting for age, sex and stage. Hazard ratios (HRs) and 95% confidence intervals (CIs) were computed based on the Cox regression analysis. Survival curves were estimated using the Kaplan–Meier method and were compared using the log-rank test. Fisher’s exact test was used to observe the differences in mortality rate and lymph node metastasis rate between different risk groups. Values of *p* < 0.05 were considered significant. All statistical analysis was performed using the R3.4.0.

## Results

### Identification of differentially methylated sites in LUAD

**Fig. 1:**
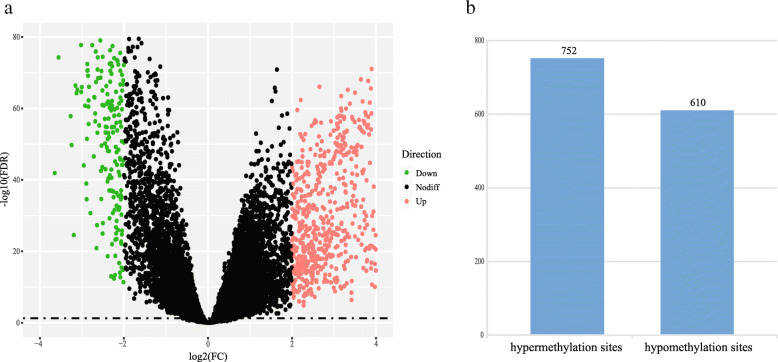
Identification of differentially expressed genes and differentially methylated sites. **a** Volcano plot of differentially expressed genes. **b** Histogram of differentially methylated sites

We initially performed differential expression analysis to select DEGs between LUAD and normal lung tissues in TCGA-LUAD dataset. With cut-off criteria of FDR < 0.05 and |log2FC| > 2.0, a total of 960 DEGs were identified, including 653 up-regulated DEGs and 307 down-regulated DEGs (Fig. 1A). We then selected the methylation sites which were differentially methylated between LUAD and normal lung tissues and significantly negatively correlated with the expression of corresponding DEGs. We thought that such methylation sites could influence the gene expression and further participate in tumor progression. The results showed that a total of 1362 DMSs corresponding to 471 DEGs were identified, including 752 hypermethylation sites and 610 hypomethylation sites (Fig. 1B).

### Functional enrichment of DMGs

**Fig. 2:**
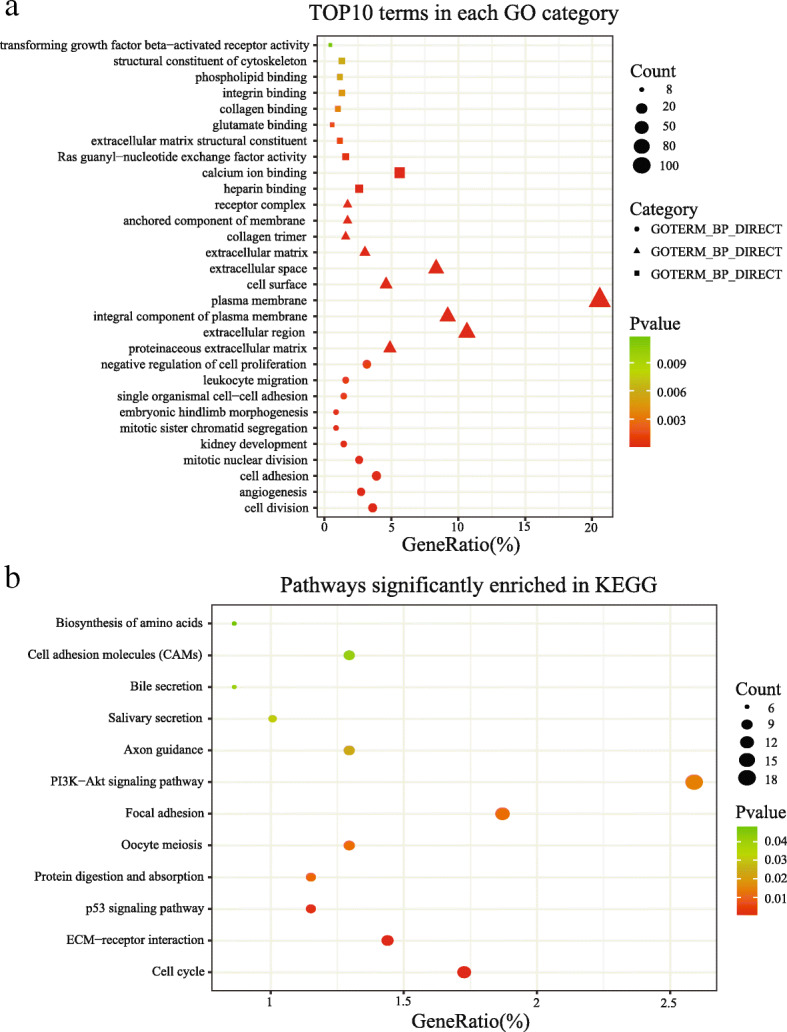
Functional enrichment of differentially expressed genes corresponding to differentially methylated sites. **a** Top ten terms significantly enriched in each Gene Ontology (GO) category. **b** Pathways significantly enriched in Kyoto Encyclopedia of Genes and Genomes (KEGG)

To further investigate the biological processes which the DMSs might be involved in, we performed GO annotation and KEGG pathway enrichment using DAVID database for the 471 corresponding DEGs. The DEGs were significantly enriched in many cancer-related pathways. The top significant terms emerging from the gene oncology enrichment analysis were shown in Fig. 2A. For instance, the most significant GO term, cell division, has been reported in multiple articles related to the progression and metastasis of cancer [15–17]. We also found that DEGs were significantly enriched in angiogenesis, which is a core hallmark of advanced cancers, especially in LUAD [18–20]. Besides, other significant GO terms, such as regulation of cell cycle and regulation of small GTPase mediated signal transduction were also related to cancer progression and chemoresistance reported in many studies [21, 22]. As shown in Fig. 2B, KEGG pathway enrichment analysis found twelve significantly enriched pathways related to cancer progression, such as PI3K-Akt signaling pathway [23, 24], ECM-receptor interaction [25, 26] and p53 signaling pathway [27, 28]. The results indicated that these DEGs played key roles in multiple cancer-related pathways, and further indicated that the DMSs might be involved in LUAD progression by regulating the corresponding gene expression.

### Construction of PPI network

**Fig. 3:**
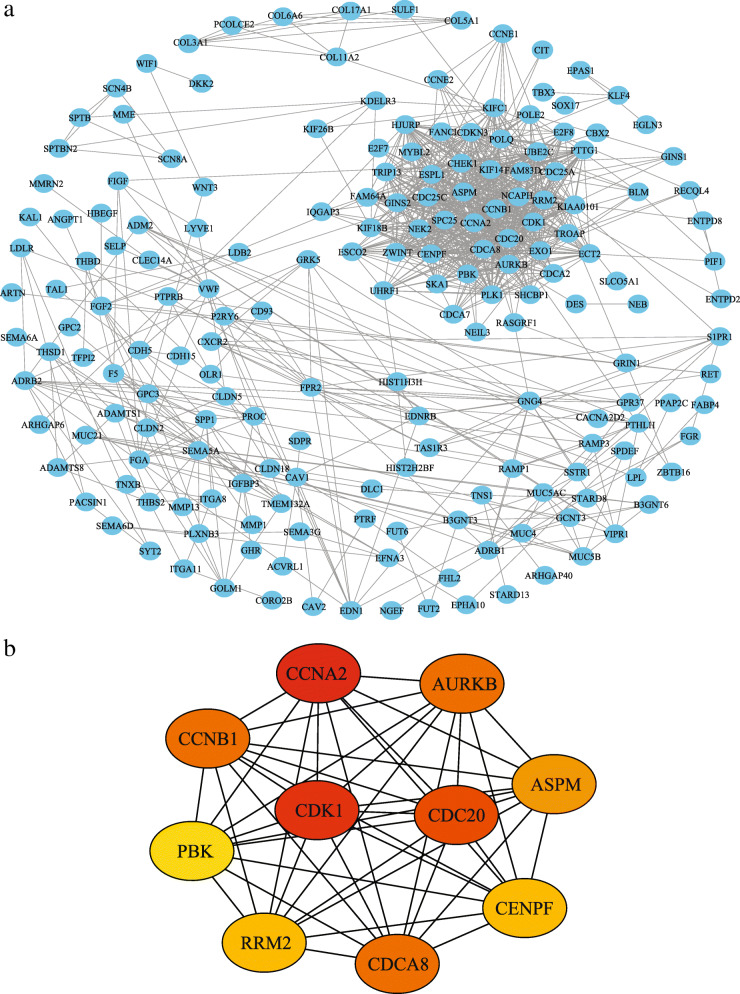
Construction of protein-protein interaction (PPI) network and Identification of hub genes. **a** PPI network. **b** Ten hub genes extracted from PPI network

Using STRING database, a PPI network was constructed to further explore the interactions between the 471 DEmRNAs. After removing unconnected nodes, the PPI network of DEGs is consisted of 188 nodes and 888 edges when combined score > 0.7 was set as the cutoff criterion (Fig. 3A). Furthermore, the top 10 hub genes, including cyclin dependent kinase 1 (CDK1), cyclin A2 (CCNA2), cyclin B1 (CCNB1), cell division cycle 20 (CDC20), cell division cycle associated 8 (CDCA8), aurora kinase B (AURKB), assembly factor for spindle microtubules (ASPM), PDZ binding kinase (PBK), ribonucleotide reductase regulatory subunit M2 (RRM2) and centromere protein F (CENPF), were identified using the cytoHubba plugin for Cytoscape, with a higher degree of connectivity (Fig. 3B). Most of ten genes had been reported to be closely related to tumorigenesis and progression of LUAD.

### Establishment of the DMSs-based prognostic signature

**Fig. 4:**
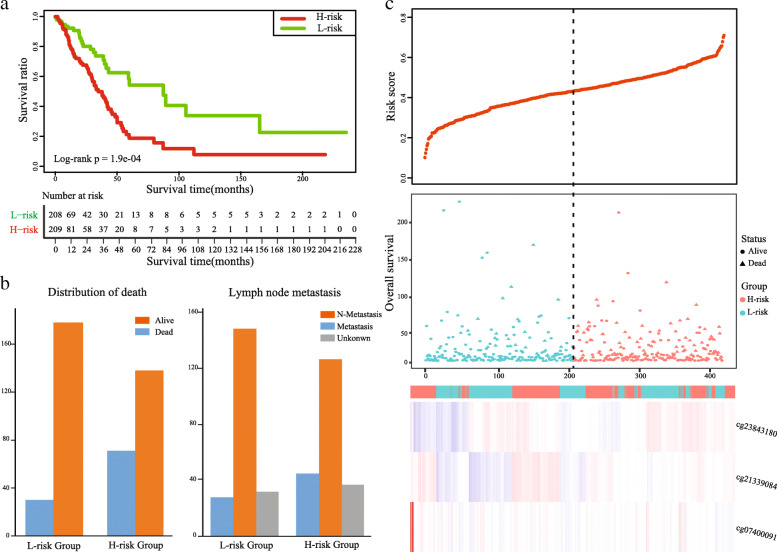
Construction of the three-DMSs prognostic signature in TCGA-LUAD cohort. **a** Kaplan-Meier curve of the overall survival for high-risk and low-risk scores ranking by the three-DMSs prognostic signature. **b** The distribution of death and lymphatic node metastasis in high-risk and low-risk groups respectively. **c** Risk score distribution, survival status and methylation heat map of three DMSs corresponding to each sample above

**Fig. 5:**
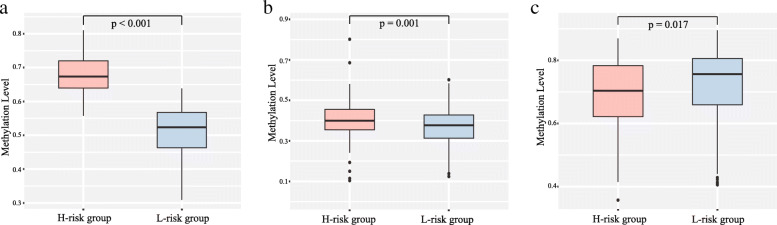
Methylation difference of three differentially methylated sites between high-risk and low-risk groups respectively. **a** cg21339084. **b** cg07400091. **c** cg23843180

Performing the univariate Cox regression analysis, we identified DMSs with potential prognostic value in TCGA-LUAD cohort. Details of the clinical characteristics are presented in Supplementary Table S1. We found that 59 DMSs were significantly associated with overall survival, including 47 hypermethylation sites and 12 hypomethylation sites. The list of 59 DMSs is showed in Supplementary Table S2. Thus, these methylation sites were defined as prognosis-related DMSs to construct the prognostic signature. We used the glmnet package in R to perform LASSO regression analysis in TCGA-LUAD cohort. We obtained the optimal value of the parameter λ, which controlled the degree of LASSO regression complexity, and selected the significant variables through multiple cross-validation. We found that the parameter λ reached the optimal value, when the number of variables was three. Therefore, combining the regression coefficients of three DMSs under the optimal λ value, we constructed a three-DMSs risk score model to guide the prognosis of LUAD patients. The general information of the three DMSs is displayed in Table 2. The risk score formula was created as follows: Risk score = (1.0003*methylation level of cg21339084) + (0.1484*methylation level of cg07400091) + (− 0.2536*methylation level of cg23843180). Calculating the risk scores for patients in TCGA-LUAD cohort, we classified patients into a high-risk or a low-risk group based on the median risk score. We found that the three-DMSs signature significantly stratified patients in terms of overall survival (log-rank *p* = 1.9E-04; Fig. 4A). Patients with high risk scores had significantly shorter OS than those with low risk scores. The mortality rate was 34.0% (71/209) in the high-risk group, significantly higher than 14.4% (30/208) in the low-risk group (*p* < 0.001, Fisher exact test; Fig. 4B). The risk score distribution, survival status, and methylation profile of the three prognostic DMSs are shown in Fig. 4C. As shown in Table 3, multivariate Cox regression analysis suggested that the three-DMSs signature was an independent prognostic factor, after adjusting for age, sex and stage (HR = 2.29, 95%CI: 1.47–3.57, *P* = 2.36E-04). Furthermore, noticing the patients with lymph node metastasis status, we found that patients in the high-risk group had a higher lymph node metastasis rate than those in the low-risk group (26.2% vs. 15.8%, *p* = 0.018, Fisher exact test; Fig. 4B). From the three DMSs, two were associated with high risk (cg21339084 and cg07400091; HR > 1) and one appeared to be protective (cg23843180; HR < 1). The methylation level of the three prognostic DMSs was detected and the differences between high- and low-risk groups were compared. We found that patients with high-risk scores tended to hypermethylation at risky sites, whereas patients in the low-risk group tended to hypomethylation at protective sites (Fig. 5A-C).

**Table 2:** General information of the three DMSs

ProbeID	Gene	chrom	chromStart	chromEnd	coefficient
cg21339084	LIMS2	chr2	128,422,432	128,422,434	1.0003
cg07400091	S1PR1	chr1	101,704,472	101,704,474	0.1484
cg23843180	NGEF	chr2	233,852,838	233,852,840	−0.2536

**Table 3:** Univariate and multivariate Cox regression analysis in TCGA-LUAD and GSE56044

Variables	Univariate analysis		Multivariate analysis	
	HR (95% CI)	*P*	HR (95% CI)	*P*
**TCGA-LUAD cohort**				
Age				
< = 60/> 60	0.96 (0.63–1.47)	0.852	1.19 (0.77–1.85)	0.437
Sex				
Male/Female	0.94 (0.64–1.40)	0.774	0.99 (0.66–1.49)	0.964
Stage				
I + II/III + IV	2.74 (1.82–4.13)	1.30e-06	2.79 (1.85–4.21)	1.06e-06
Risk score				
Low/High	2.22 (1.45–3.42)	2.77e-04	2.29 (1.47–3.57)	2.36e-04
**GSE56044 cohort**				
Age				
< = 60/> 60	2.83 (1.26–6.35)	0.012	2.88 (1.26–6.59)	0.012
Sex				
Male/Female	1.10 (0.63–1.93)	0.737	0.79 (0.44–1.43)	0.438
Risk score				
Low/High	2.15 (1.20–3.85)	0.010	2.16 (1.19–3.91)	0.011

### Prognostic validation of the three-DMSs signature

**Fig. 6:**
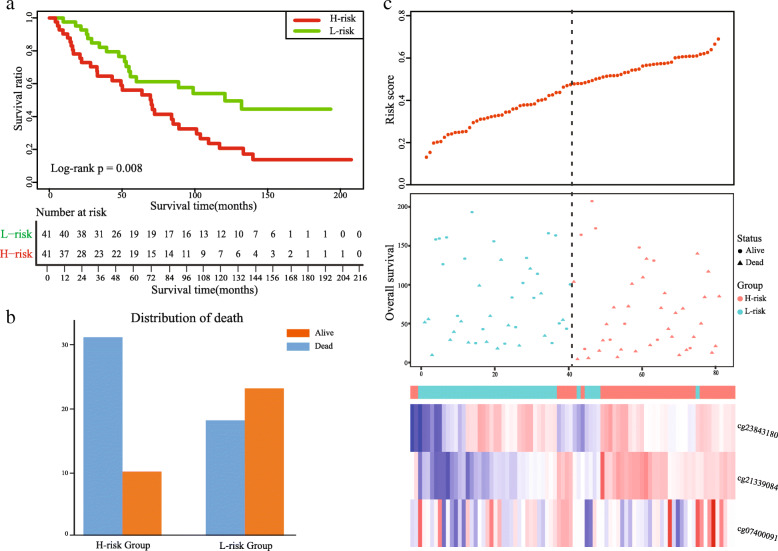
Validation of the three-DMSs prognostic signature in an independent cohort. **a** Kaplan-Meier curve of the overall survival for high-risk and low-risk scores ranking by the three-DMSs prognostic signature. **b** The distribution of death in high-risk and low-risk groups. **c** Risk score distribution, survival status and methylation heat map of three DMSs corresponding to each sample above

An independent cohort (GSE56044), containing 82 LUAD patients with both methylation data and clinical information, was used to validate the prognosis performance of the three-DMSs signature. Similarly, we calculated the risk score for each patient using the three-DMSs signature, after which patients were classified into a high-risk (*n* = 41) or a low-risk (*n* = 41) group based on the median risk score. We found that patients in high-risk group had a shorter survival time than those in low-risk group (HR = 2.15, 95% CI: 1.20–3.85, log-rank *p* = 0.008, Fig. 6A). Furthermore, we calculated the mortality rate in each risk group. The result showed that the mortality rate in high-risk group was 32% higher than that in low-risk group (*p* = 0.006, Fisher exact test; Fig. 6B). The risk score distribution, survival status, and expression profile of the three prognostic DMSs are shown in Fig. 6C. As biased stage information, the stage variable is excluded when performed multivariate Cox regression analysis. In accordance with the result of training set, the multivariate Cox regression analysis confirmed that the three-DMSs signature was significantly correlated with overall survival as an independent prognostic factor (HR = 2.16, 95% CI: 1.19–3.91, *P* = 0.011, Table 3).

### Construction of three-DMSs signature-based nomogram

**Fig. 7:**
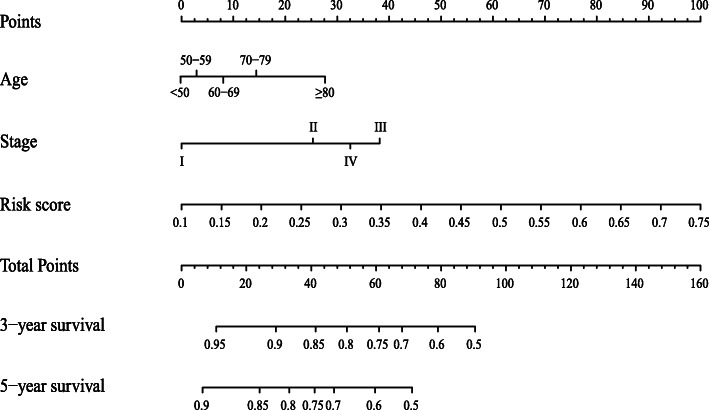
A nomogram for the prediction of 3- and 5-years overall survival in LUAD patients

**Fig. 8:**
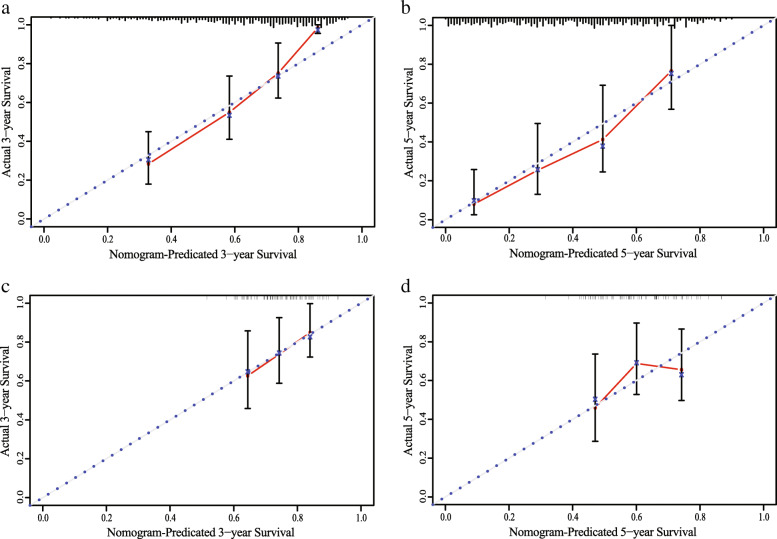
Calibration curves of the nomogram for 3- and 5-years overall survival predictions in TCGA-LUAD cohort (**a**,**b**) and GSE56044 cohort (**c**,**d**)

Multivariate Cox analysis indicated that three variables (age, stage, and three-DMSs risk score) were independent risk factors for OS. Thus, a nomogram predicting 3- and 5-years OS was constructed based on the multivariate analysis data. As shown in Fig. 7, the total points for a patient can be obtained by adding the points from each independent prognostic factor listed in the nomogram. C-indexes for the nomogram were 0.71 (95%CI: 0.58–0.85) and 0.70 (95%CI: 0.52–0.88) in TCGA-LUAD and GSE56044 cohorts, respectively. The calibration plots for the probabilities of 3 and 5-year OS indicated no apparent departure from the ideal line, showing good agreement between the nomogram-predicted OS and actual OS of LUAD patients in both the training and validation cohorts (Fig. 8). Such results indicated that the three-DMSs signature-based nomogram could provide insight into regarding survival prediction and serve as a clinically available guide for personalized treatment of LUAD patients.

## Discussion

Due to the heterogeneity of LUAD, it is still a great challenge to develop successful individual-based treatment [29, 30]. Aberrant DNA methylation is of considerable importance in LUAD onset and progression [31, 32]. A special focus on DNA methylation alterations to develop the prognostic and predictive signatures for LUAD patients would be meaningful for survival prediction, guiding the personalized treatment decisions. Zheng et al. [11] constructed a CpG-based signature for survival prediction of lung adenocarcinoma patients based on TCGA database. However, such studies were limited by either small sample size or lack of validation of the signature as an independent prognostic factor. Therefore, in-depth studies on the LUAD progressive mechanisms, identification of specific methylation CpG sites and construction of the robust prognostic signatures are urgently required.

In the present study, we screened the DMSs that significantly correlated with corresponding gene expression, which may be involved in cancer progression by regulating the gene expression. Thus, a three-DMSs methylation signature significantly associated with the OS of LUAD patients was constructed based on genome-wide DNA methylation profiles using the Cox regression and LASSO analyses. The three-DMSs signature performed well in classifying patients into a high-risk or a low-risk group with significant survival difference. Furthermore, a nomogram was developed by combing the DMSs-based prognostic signature with clinical risk factors, which could provide a clinically available and robust guide for survival prediction and personalized treatment of LUAD patients.

Our study showed that three DMSs within prognostic signature had a critical role in progression and metastasis of LUAD. The three DMSs, including cg21339084, cg07400091 and cg23843180, correspond to LIMS2, S1PR1 and NGEF respectively (Table S3). We found that cg21339084 and cg07400091 were located in the S_Shore of CpG islands. The hypermethylation of cg21339084 and cg07400091 was significantly correlated with loss of expressions of LIMS2 and S1PR1. The cg23843180 was located in the 5’UTR of promoter, whose hypomethylation increased the expression of NGEF. Beside, we found that all three DMSs were located in DNase-I-hypersensitive sites (DHS) region, indicting the relationship between DNA methylation and DHS. Furthermore, we annotated all methylation probes of the three genes, and calculated the methylation difference and correlation with gene expression. The results showed that almost all 36 probes of LIMS2 were located in the promoter region and were hyper-methylated, indicating that loss of expressions of LIMS2 was significantly affected by promoter methylation. Many researches had demonstrated that frequent epigenetic silencing of LIMS2 could be important in GC progression events [33]. A total of 21 methylation probes of S1PR1 were located around the CpG islands. All probes were significantly hyper-methylated in LUAD samples except cg10020333, indicating that the hypermethylation of S1PR1 was closely related to LUAD progression. Previous study had shown that S1PR1 could act as methylation-driven genes to reveal prognostic biomarkers in LUAD [34]. Besides, 21 methylation probes were annotated within NGEF. NGEF is a novel member of the family of Dbl genes and functions as a guanine nucleotide exchange factor for the Rho-type GTPases. Few studies described its roles in carcinogenesis [35]. We found that the distributions and methylation levels of these probes were different, indicating that there might be multiple regulatory mechanisms in LUAD progression. These results indicated the aberrant methylation of three DMSs might play vital roles in promoting LUAD progression and metastasis, but the underlying mechanisms need further experimental verification.

In this study, we selected methylation sites that were significantly negatively correlated with the expression to ensure the regulatory effect on genes. However, several important methylation sites might be lost due to lack of significance. The expression of genes is regulated by lots of factors rather than methylation, such as mutation and copy number variation. For example, cg26500801 was located in CpG island of KEAP1. We found that cg26500801 was significantly hyper-methylated in LUAD samples. Previous research had confirmed the effect of methylation on KEAP1 transcription control across multiple histologies of lung cancer [36]. However, we found that the correlation between methylation level of cg26500801 and expression level of KEAP1 is not significant. We observed that 17 % of samples had KEAP1 mutations. Recent studies demonstrated that KEAP1/NRF2 axis dysfunction is strongly related to tumor progression and chemo- and radiotherapy resistance of cancer cells [37]. Fabrizio et al. reported that epigenetic abnormalities were demonstrated as emerging mechanisms of KEAP1/NRF2 axis modulation in addition to the most frequently investigated point mutations in solid tumors [38]. Elshaer et al. also found that KEAP1 mutations were associated with DNA methylation changes capable of shaping regulatory network functions [39]. Similarly, cg00912625 is a methylation site that is located in CpG island of CNTN4. Our results showed that cg00912625 was also significantly hyper-methylated in LUAD samples. However, the correlation between methylation level of cg00912625 and expression level of CNTN4 is not significant. We found that CNTN4 loss accounted for distinctly higher proportion than its gain in LUAD samples, indicating that CNV might contribute to abnormal expression. Therefore, combining both epigenomic and transcriptomic changes along with genetic alterations may provide a better understanding of the molecular mechanisms associated with the progression of lung cancer and may help to provide better therapeutic approaches.

## Conclusion

Analyzing methylation and expression data comprehensively, our study identified a robust three-DMSs prognostic signature, which was significantly associated with the OS of LUAD patients. Furthermore, a nomogram was developed by combing the three-DMSs prognostic signature with clinical risk factors, which could provide a clinically available and robust guide for survival prediction and personalized treatment of LUAD patients. Further studies on the functional mechanism of the three DMSs could be carried out, which might provide helpful guidance for LUAD therapy as promising therapeutic targets in the near future.

## Supplementary Information

**Additional file 1: Table S1.** Clinical information analyzed in present study.

**Additional file 2: Table S2.** The list of prognosis-related DMSs.

**Additional file 3: Table S3.** Annotation of methylation probes corresponding to three target genes.

## Data Availability

All data generated or analyzed during this study are included in this published article.

## Abbreviations

LUAD: Lung adenocarcinoma
GEO: Gene expression omnibus
TCGA: The cancer genome atlas
DMSs: Differentially methylated sites
DEGs: Differentially expressed genes
GO: Gene oncology
KEGG: Kyoto encyclopaedia of genes and genomes
LASSO: The least absolute shrinkage and selection operator
OS: Overall survival
HR: Hazard ratios
CI: Confidence interval
