# Supplementary material for: A methylation-based nomogram for predicting survival in patients with lung adenocarcinoma
Source: BMC Cancer. 2021 Jul 12;21:801. doi: 10.1186/s12885-021-08539-4 (PMC8273993; doi:10.1186/s12885-021-08539-4)
Supplement: Supplementary file 1 — Additional file 1: Table S1. Clinical information analyzed in present study. [file 12885_2021_8539_MOESM1_ESM.docx]

Table S1 Clinical information analyzed in present study

|  | **Training cohort**  **(TCGA-LUAD)** | **Validation cohort**  **(GSE56044)** |
| --- | --- | --- |
| **Sample** |  |  |
| Normal | - | - |
| Tumor | 417 | 82 |
| **Mean age**  (years;range) | 65  (33-88) | 66  (36-83) |
| **Sex** |  |  |
| Male | 193 | 35 |
| Female | 224 | 47 |
| **Stage** |  |  |
| I | 229 | - |
| II | 102 | - |
| III | 66 | - |
| IV | 20 | - |
| **Status** |  |  |
| Alive | 101 | 33 |
| Dead | 316 | 49 |
