# Supplementary material for: A methylation-based nomogram for predicting survival in patients with lung adenocarcinoma
Source: BMC Cancer. 2021 Jul 12;21:801. doi: 10.1186/s12885-021-08539-4 (PMC8273993; doi:10.1186/s12885-021-08539-4)
Supplement: Supplementary file 2 — Additional file 2: Table S2. The list of prognosis-related DMSs. [file 12885_2021_8539_MOESM2_ESM.docx]

Table S2 List of prognosis-related DMSs

|  | Probe ID |
| --- | --- |
| Hypermethylation | cg18044111 cg13758712 cg01069941 cg08390172 cg25266895 cg22505962 cg15384383 cg15333318 cg04316429 cg24936799 cg12339905 cg06638451 cg21127268 cg07234508 cg03075966 cg13505393 cg06117855 cg12033622 cg07229186 cg03229627 cg15258447 cg16953473 cg21339084 cg20742009 cg02757194 cg10227358 cg25481157 cg07400091 cg09827761 cg24030622 cg14119581 cg05008975 cg20031656 cg06422678 cg01302668 cg04816699 cg14610066 cg09489462 cg10324158 cg03725573 cg03382304 cg27019278 cg24622589 cg25224568 cg07262244 cg24763243 cg15623503 |
| Hypomethylation | cg00715835 cg21693965 cg01850449 cg04474257 cg22069262 cg00088885 cg09622982 cg15665400 cg23979520 cg08300252 cg23843180 cg11837910 |
